# Impact of reduced dietary calcium and phosphorus levels on nutrient digestibility, metabolic homeostasis, and immune responses in weaned piglets

**DOI:** 10.3389/fvets.2026.1821939

**Published:** 2026-05-29

**Authors:** Xiurong Peng, Jin Chen, Yanan Liu, Siyu Xiong, Jiabao Yang

**Affiliations:** 1 Sichuan Academy of Animal Sciences, Chengdu, China; 2 Animal Genetic Breeding and Reproduction Key Laboratory of Sichuan Province, Chengdu, China

**Keywords:** Calcium, growth performance, nutrient digestibility, phosphorus, weaned piglets

## Abstract

This study investigated the effects of dietary calcium (Ca) and phosphorus (P) levels on growth performance, diarrhea incidence, blood parameters, serum biochemical indices, nutrient digestibility, and antioxidant and immune functions in weaned piglets. A total of 120 healthy piglets (initial body weight: 7.99 ± 1.15 kg) were randomly allocated to two treatment groups: a high Ca–P diet (HCP: Ca 0.85%, P 0.70%) and a low Ca–P diet (LCP: Ca 0.60%, P 0.60%). Each treatment had six replicates with ten piglets per replicate, and the experimental period lasted 39 days. Compared with the high Ca–P group, the low Ca–P group showed partially increased average daily gain (ADG) and average daily feed intake (ADFI) and a reduced feed-to-gain ratio (F/G), but the differences were not significant (*P* > 0.05). No significant difference in diarrhea incidence was observed between the two groups (*P* > 0.05). The apparent digestibility of crude protein, crude fiber, crude ash, calcium, and total phosphorus in the low Ca–P group was significantly higher than that in the high Ca–P group (*P* < 0.05). Compared with the high Ca–P group, serum aspartate aminotransferase (AST) activity in the low Ca–P group was reduced by 48.16% (*P* < 0.05), while white blood cell and monocyte counts and serum interleukin-10 (IL-10) and interferon-γ (IFN-γ) concentrations were significantly decreased (*P* < 0.05); in contrast, serum alkaline phosphatase (ALP) activity increased by 27.45% (*P* < 0.05). In summary, reducing dietary Ca and P levels to 0.60% improved nutrient digestibility, alleviated hepatic metabolic stress, and reduced basal immune activation in weaned piglets. These results provide useful insights for optimizing dietary mineral supply during the early weaning period. A primary limitation of this study is that only two Ca-P levels were tested, and bone traits, and long-term performance were not evaluated.

## 1 Introduction

Calcium (Ca) and phosphorus (P) are the most abundant mineral elements in piglet diets and play essential roles in skeletal development, energy metabolism, cellular signal transduction, and the maintenance of acid–base balance (1–3). Weaned piglets exhibit rapid growth and therefore have high requirements for Ca and P. However, due to the low bioavailability of phytate-bound phosphorus, conventional piglet diets often contain excessive levels of Ca and P to ensure adequate bone development (4). Such over-supplementation not only increases feed costs but also leads to excessive phosphorus excretion, which is a major contributor to environmental pollution such as eutrophication (4, 5). Consequently, improving the utilization efficiency of dietary Ca and P through precision nutrition strategies has become an important focus in modern pig nutrition research.

Although recent studies have primarily focused on improving phytate phosphorus utilization through phytase supplementation (4, 6, 7), the role of dietary Ca levels has often been underestimated. Evidence suggests that even in the presence of phytase, excessive Ca may act as an antinutritional factor and exert adverse effects on piglet health (8). High dietary Ca can impair digestion through multiple mechanisms: it readily forms insoluble complexes with phytate, thereby reducing the digestibility of protein, fat, and minerals (9), and it can also bind with fatty acids to form calcium soaps, which decreases fat utilization efficiency (10). Therefore, it is necessary to evaluate whether moderately reducing dietary Ca levels, while ensuring adequate *P* supply, can alleviate these antagonistic effects.

Most existing studies on dietary Ca and P optimization in weaned piglets have focused on growth performance and bone traits (11, 12), whereas comprehensive assessments of nutrient digestibility, metabolic homeostasis, and immune status remain limited. In particular, changes in circulating immune cells and cytokines may serve as sensitive indicators of basal inflammatory load and energy allocation. Accordingly, this study compared a Ca–P regimen exceeding recommended levels (0.85%/0.70%) with a more appropriate regimen (0.60%/0.60%) to evaluate the effects of reduced dietary Ca and P on growth performance, nutrient utilization, serum biochemical parameters, and immune function in weaned piglets. The results aim to provide updated evidence for the formulation of more precise and environmentally responsible mineral nutrition strategies during early weaning.

## 2 Materials and methods

All animal experiments were approved by the Ethics Review Committee of Sichuan Academy of Animal Sciences.

### 2.1 Experimental design and diets

A total of 120 healthy weaned piglets (initial body weight: 7.99 ± 1.15 kg) were selected and randomly assigned to two dietary treatments based on similar initial body weight and sex ratio. Each treatment consisted of six replicates with ten piglets per replicate. Piglets were randomly allocated to a control group representing conventional commercial practice (high Ca-P diet, HCP: Ca 0.85%, P 0.70%) and an experimental group (low Ca-P diet, LCP: Ca 0.60%, P 0.60%). The basal diet was corn–soybean meal–based, and the ingredient composition and calculated nutrient levels are presented in Table 1. The pen was considered the experimental unit for growth performance, feed intake, diarrhea incidence, and apparent nutrient digestibility. For hematological, serum biochemical, antioxidant, and immune parameters, one piglet was randomly selected from each pen, and the individual piglet was considered the experimental unit.

**Table 1:** Composition and nutritional levels of the experimental diets (air-dry basis).

Ingredients %	HCP	LCP	Nutrient level	HCP	LCP
Corn	66.45	66.45	Digestible energy (Mcal/kg)	3.30	3.30
Soybean meal	25.00	25.00	Crude Protein/%	17.8	17.8
Limestone	0.80	0.40	Calcium/%	0.85	0.6
Dicalcium phosphate	2.00	1.60	Total phosphorus/%	0.7	0.6
zeolite	0.50	1.30	Available phosphorus/%	0.46	0.4
Pre mixed material	5.25	5.25	D-Lysine/%	1.24	1.24
Total	100	100	D-Methionine/%	0.44	0.44
			D-Methionine^+^ D-Cystine/%	0.68	0.68
			D-Threonine/%	0.74	0.74
			D-Tryptophan/%	0.2	0.2

The premix includes salt, lysine hydrochloride, methionine, threonine, tryptophan and trace elements, vitamins, phytase, compound acidifiers, providing 110 mg/kg of copper, 100 mg/kg of iron, 25 mg/kg of manganese, 100 mg/kg of zinc, 0.3 mg/kg of selenium, and 0.6 mg/kg of iodine per kg of diet mg/kg, VA 13000IU/kg, VD_3_ 3500 IU/kg, VE 30 mg/kg, VK 34.8 mg/kg, VB_12_ 0.048 mg/kg, VB_1_ 4 mg/kg, VB_2_ 11 mg/kg, VB_6_ 5.2 mg/kg, Niacinamide 40 mg/kg, pantothenic acid 21 mg/kg, folic acid 2 mg/kg, biotin 0.2 mg/kg, phytase 1500 u/kg.

### 2.2 Animal management

The experiment was conducted at a commercial pig farm in Santai County, Mianyang City, Sichuan Province, China, and lasted for 39 days. Piglets had *ad libitum* access to feed and water throughout the experiment. Routine management practices and immunization programs were implemented according to farm protocols. Feed intake, health status, fecal consistency, and ambient temperature were recorded daily.

### 2.3 Sample collection

#### 2.3.1 Blood sampling

On day 12 of the experiment at 06:00 h, one piglet per replicate was randomly selected for blood collection. Approximately 5 mL of blood was collected from the anterior vena cava, centrifuged at 2,000 r/min for 3 min to obtain serum, and stored at −20°C for subsequent analysis. An additional 5 mL of EDTA-K3–anticoagulated blood was collected for hematological and flow cytometric analyses.

#### 2.3.2 Fecal sampling

Fecal samples were collected during the last 3 days of the experiment. Fresh feces were collected immediately after defecation, pooled by pen, and approximately 300 g was retained. Samples were acidified with 10% HCl (5 mL/100 g feces), supplemented with a small amount of toluene, and stored at −20°C. Prior to analysis, fecal samples were dried at 60°C for 48 h, ground, passed through a 40-mesh sieve, and stored at 4°C.

### 2.4 Measurements and analytical methods

#### 2.4.1 Growth performance

Piglets were weighed after overnight fasting at the beginning and end of the experiment. Feed intake was recorded weekly by replicate to calculate ADG, ADFI, and F/G.

#### 2.4.2 Diarrhea incidence

Fecal consistency was evaluated three times daily and scored as follows: 1 = soft feces; 2 = pasty feces; 3 = watery feces. Piglets with scores ≥ 2 were considered diarrheic. Diarrhea rate and diarrhea index were calculated accordingly.

Diarrhea frequency (%) = total number of diarrhea pigs/(number of experimental pigs x days) x 100;

Diarrhea index = sum of diarrhea scores/number of experimental pigs.

#### 2.4.3 Hematological, biochemical, and immune indices

Hematological parameters were analyzed using an automated hematology analyzer (IDEXX, USA). CD4^+^ and CD8^+^ T lymphocyte proportions were determined by flow cytometry (Beckman Coulter, USA). Serum biochemical indices, immune cytokines, and antioxidant parameters were measured using commercial kits according to the manufacturers' instructions.

#### 2.4.4 Apparent nutrient digestibility

Acid-insoluble ash was used as an endogenous marker to determine the apparent digestibility of nutrients, including crude protein, crude fat, crude fiber, crude ash, calcium, and total phosphorus. The calculation formula for apparent nutrient digestibility is as follows:

Nutrient digestibility = [1 – (indicator (acid insoluble ash) content in feed x nutrient content in feces)/(indicator content in feces (acid insoluble ash) x nutrient content in feed)] x 100.

### 2.5 Statistical analysis

Data were initially processed using Excel 2007 and analyzed using SPSS 18.0. Because only two dietary treatments were compared, continuous variables were analyzed using independent-sample *t*-tests to determine treatment effects. Diarrhea incidence and other categorical or proportional data were analyzed using chi-square tests when appropriate. The pen was used as the experimental unit for growth performance, feed intake, diarrhea incidence, and apparent nutrient digestibility, whereas the individual piglet was used as the experimental unit for blood, serum biochemical, antioxidant, and immune parameters. Results are presented as mean ± standard deviation. Statistical significance was declared at *P* < 0.05, and *P* < 0.01 was considered highly significant.

## 3 Results

### 3.1 Growth performance and diarrhea incidence

As shown in Table 2, reducing dietary calcium and phosphorus levels from 0.85%/0.70% to 0.60%/0.60% had no significant effects on average daily gain (ADG), average daily feed intake (ADFI), feed-to-gain ratio (F/G), or diarrhea-related indices in weaned piglets throughout the experimental period (*P* > 0.05). Although no statistically significant differences were detected, piglets fed the low Ca–P diet exhibited numerically higher ADG and ADFI, with increases of 4.75 and 4.63%, respectively, compared with those fed the high Ca–P diet. Diarrhea index and diarrhea incidence did not differ between dietary treatments.

**Table 2:** Effects of dietary calcium and phosphorus levels on piglet growth performance and diarrhea.

Item	HCP	LCP	*P*-value
IBW (kg)	7.981 ± 0.30	7.991 ± 0.11	0.991
FBW (kg)	28.885 ± 0.28	30.132 ± 0.73	0.618
ADG (g/d)	535.681 ± 02.92	567.734 ± 8.50	0.506
ADFI (g/d)	916.511 ± 31.39	960.066 ± 4.59	0.634
FCR	1.710 ± 0.08	1.690 ± 0.04	0.629
Diarrhea Index	4.022 ± 0.21	5.231 ± 0.65	0.305
Diarrhea rate (%)	5.252 ± 0.87	7.042 ± 0.09	0.245

IBW, initial body weight; FBW, final body weight; ADG, average daily gain; ADFI, average daily feed intake; FCR, feed conversion rate.

### 3.2 Nutrient apparent digestibility

As shown in Table 3, LCP significantly increased the apparent digestibility of several nutrients. Compared with HCP, LCP increased crude protein digestibility from 75.94 to 78.25% (*P* = 0.032), crude ash digestibility from 52.81% to 55.48% (*P* = 0.034), crude fiber digestibility from 29.59 to 43.04% (*P* = 0.005), total phosphorus digestibility from 59.02 to 61.74% (*P* = 0.048), and calcium digestibility from 61.10 to 66.25% (*P* = 0.005). No significant difference was observed in crude fat digestibility between treatments (*P* > 0.05).

**Table 3:** The impact of calcium and phosphorus levels on the digestibility of nutrients by piglets.

Item (%)	HCP	LCP	*P*-value
CP	75.941 ± 0.59	78.251 ± 0.64	0.032
CA	52.811 ± 0.64	55.482 ± 0.10	0.034
CF	29.595 ± 0.57	43.047 ± 0.46A	0.005
EE	74.462 ± 0.47	73.012 ± .82	0.366
TP	59.022 ± 0.21	61.741 ± 0.98	0.048
Calcium	61.102 ± 0.42	66.252 ± 0.62	0.005

CP, crude protein; CA, crude ash; CF, crude fiber; EE, ether extract; TP, total phosphorus.

### 3.3 Hematological parameters

As shown in Table 4, LCP significantly reduced total white blood cell and monocyte counts. White blood cell count decreased from 19.11 × 10^9^/L in HCP to 15.90 × 10^9^/L in LCP (*P* = 0.039). Monocyte count decreased from 2.75 × 10^9^/L to 1.42 × 10^9^/L (*P* = 0.001). No significant differences were observed in lymphocyte, neutrophil, eosinophil, basophil, red blood cell, hemoglobin, or platelet counts between treatments.

**Table 4:** The effects of calcium and phosphorus levels on blood routine indicators of weaned piglets.

Item	HCP	LCP	*P*-value
White blood cell count ( × 10^9^/L)	19.110 ± 0.52	15.902 ± 0.00	0.039
Lymphocyte count ( × 10^9^/L)	10.111 ± 0.05	9.332 ± 0.20	0.596
Monocyte count ( × 10^9^/L)	2.750 ± 0.45	1.420 ± 0.23	0.001
Neutrophil count ( × 10^9^/L)	6.120 ± 0.97	5.001 ± 0.18	0.220
Eosinophil count ( × 10^9^/L)	0.110 ± 0.05	0.120 ± 0.04	0.808
Eosinophil Count ( × 10^9^/L)	0.010 ± 0.01	0.010 ± 0.02	0.946
Red blood cell count ( × 1,012/L)	6.680 ± 0.40	6.370 ± 0.59	0.459
Hemoglobin content (g/dL)	10.901 ± 0.14	10.000 ± 0.85	0.243
Platelet count ( × 10^3^/μL)	342.671 ± 00.98	336.404 ± 7.56	0.907

### 3.4 Serum biochemical parameters

As shown in Table 5, serum calcium and inorganic phosphorus concentrations did not differ between dietary treatments (*P* > 0.05). Serum alkaline phosphatase (ALP) activity was significantly higher in piglets fed the low Ca–P diet than in those fed the high Ca–P diet (*P* = 0.038). In contrast, serum aspartate aminotransferase (AST) activity was significantly lower in the low Ca–P group (*P* = 0.028). No significant differences were detected in alanine aminotransferase (ALT), glucose, total protein, albumin, globulin, triglyceride, or urea concentrations between treatments (*P* > 0.05).

**Table 5:** Effects of calcium and phosphorus levels on serum biochemical indicators of weaned piglets.

Item	HCP	LCP	*P*-value
Calcium (mmol/L)	2.580 ± 0.13	2.700 ± 0.20	0.256
Phosphorus (mmol/L)	3.450 ± 0.30	3.430 ± 0.23	0.909
ALP (U/L)	377.677 ± 8.63	481.337 ± 1.31	0.038
ALT (U/L)	89.733 ± 1.89	84.931 ± 5.92	0.748
AST (U/L)	89.253 ± 5.80	46.271 ± 9.77	0.028
Glucose (mmol/L)	6.300 ± 0.44	6.160 ± 0.54	0.627
Total protein (g/L)	55.655 ± 0.03	51.723 ± 0.55	0.149
Albumin (g/L)	31.574 ± 0.66	30.782 ± 0.07	0.715
Triglycerides (mmol/L)	0.450 ± 0.20	0.480 ± 0.15	0.835
Urea (mmol/L)	1.741 ± 0.03	1.280 ± 0.42	0.338
Globulin (g/L)	24.083 ± 0.45	20.933 ± 0.22	0.133

### 3.5 Serum antioxidant parameters

The serum antioxidant indices are presented in Table 6. Dietary Ca and P levels had no significant effects on the activities of glutathione peroxidase (GSH-Px), total superoxide dismutase (T-SOD), or catalase (CAT), nor on total antioxidant capacity (T-AOC) or malondialdehyde (MDA) concentration (*P* > 0.05).

**Table 6:** The effects of calcium and phosphorus levels on antioxidant indexes in the serum of weaned piglets.

Item	HCP	LCP	*P*-value
GSH-Px (U/mL)	305.872 ± 4.06	297.543 ± 9.40	0.718
T-SOD (U/mL)	77.084 ± 0.06	78.543 ± 0.70	0.586
MDA (nmol/mL)	3.751 ± 0.55	4.342 ± 0.21	0.657
T-AOC (mM)	0.220 ± 0.03	0.260 ± 0.09	0.375
CAT (U/mL)	7.062 ± 0.12	5.572 ± 0.07	0.303

### 3.6 Serum immune parameters and lymphocyte subsets

**Figure 1:**
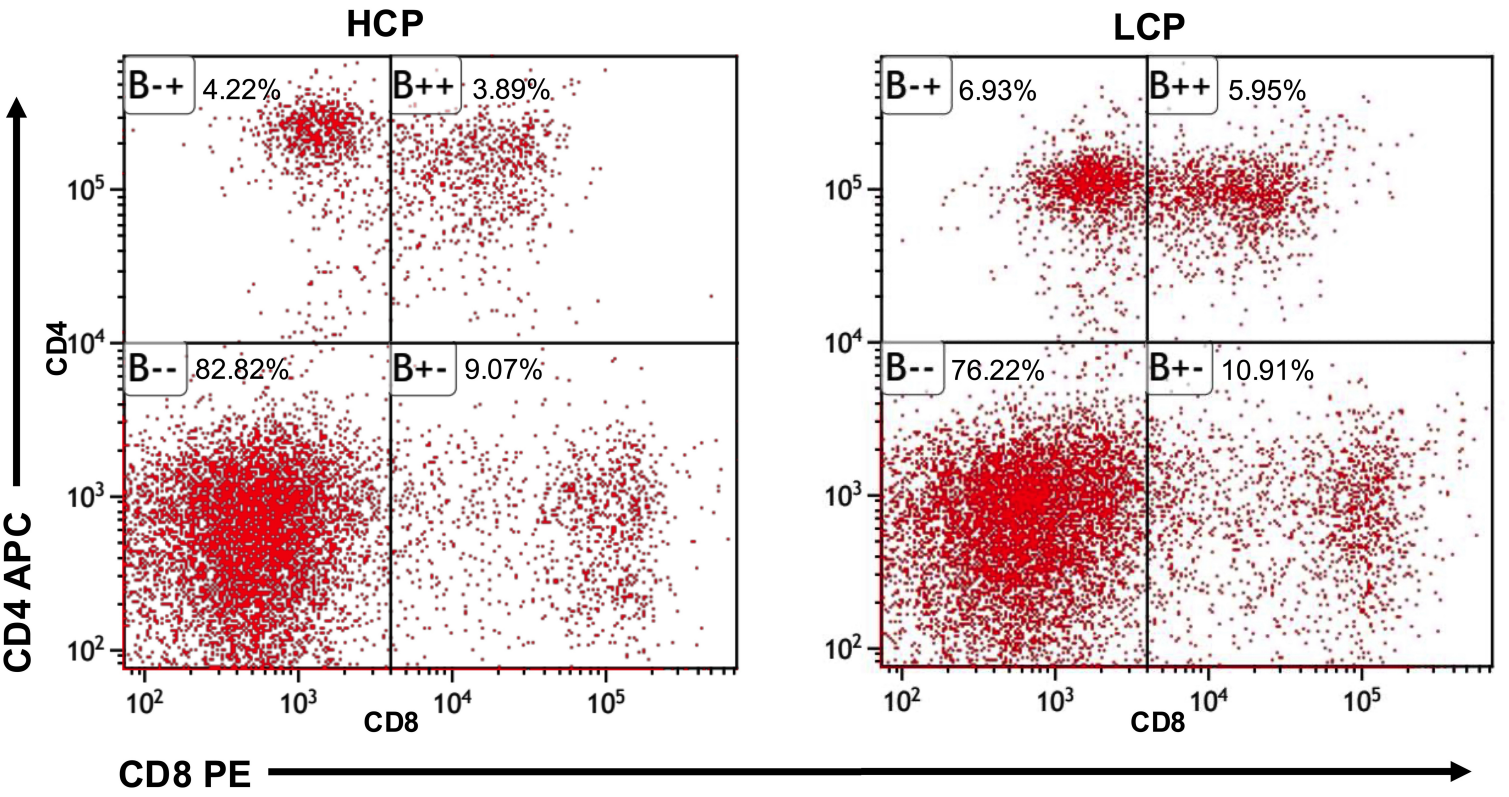
Flow cytometry analysis of the effects of dietary calcium and phosphorus levels on the proportions of CD4^+^ and CD8^+^ lymphocytes and the CD4^+^/CD8^+^ ratio in peripheral blood of piglets.

As shown in Table 7, piglets fed the low Ca–P diet exhibited significantly lower serum concentrations of interleukin-10 (IL-10; *P* = 0.048) and interferon-γ (IFN-γ; *P* = 0.002) compared with those fed the high Ca–P diet. Serum IL-2 and tumor necrosis factor-α (TNF-α) concentrations tended to decrease in the low Ca–P group, although the differences were not statistically significant (*P* > 0.05). No significant differences were observed in immunoglobulin (IgA, IgG, and IgM) concentrations between treatments. The proportions of CD4^+^ and CD8^+^ T lymphocytes and the CD4^+^/CD8^+^ ratio in peripheral blood are shown in Table 8 and Figure 1. No significant differences were detected between dietary treatments (*P* > 0.05).

**Table 7:** The effects of calcium and phosphorus levels on serum immune indicators of weaned piglets.

Item	HCP	LCP	*P*-value
Ig A (μ g/mL)	727.964 ± 5.73	672.694 ± 6.90	0.103
Ig G (mg/mL)	24.830 ± 0.84	23.192 ± 0.26	0.210
Ig M (mg/mL)	19.202 ± 0.15	17.761 ± 0.76	0.277
IL-1 β (pg/mL)	534.915 ± 2.60	516.044 ± 6.99	0.569
IL-2 (pg/mL)	286.012 ± 4.20	256.071 ± 9.40	0.061
IL-6 (pg/mL)	924.245 ± 6.85	891.161 ± 01.22	0.573
IL-10 (pg/mL)	177.255 ± 0.92	165.109 ± 0.11	0.048
C-reactive protein (mg/L)	11.811 ± 0.08	11.650 ± 0.87	0.806
TNF alpha (pg/mL)	193.801 ± 0.86	178.751 ± 3.21	0.096
IFN-γ (pg/mL)	41.631 ± 0.41	34.862 ± 0.72	0.002
Lysozyme (μ g/mL)	38.184 ± 0.09	34.852 ± 0.74	0.158

**Table 8:** Effects of calcium and phosphorus levels on the proportions and ratio of CD4^+^ and CD8^+^ lymphocytes in peripheral blood of piglets.

Item	HCP	LCP	*P*-value
CD4^+^ %	8.323 ± 0.87	12.924 ± 0.06	0.166
CD8^+^ %	12.746 ± 0.05	17.235 ± 0.84	0.339
CD4^+^/CD8^+^	0.650 ± 0.16	0.760 ± 0.13	0.317

## 4 Discussion

In the present study, reducing dietary Ca and P levels to 0.60%/0.60% did not impair growth performance or increase diarrhea incidence in weaned piglets. These results indicate that the lower Ca–P regimen was sufficient to support normal growth during the post-weaning period. Similar findings have been reported previously, suggesting that Ca and P levels exceeding actual physiological requirements do not necessarily confer additional growth benefits and may even limit performance by reducing nutrient availability (11, 12). The numerical improvements observed in ADG and ADFI further suggest that excessive Ca and P supplementation is not required to maintain optimal growth in weaned piglets when diets are otherwise nutritionally adequate.

One of the most notable findings of this study was the significant improvement in apparent nutrient digestibility following the reduction of dietary Ca and P levels. Excessive dietary Ca is known to form insoluble complexes with phytate, thereby decreasing the availability of phosphorus and entrapping other nutrients such as proteins and carbohydrates (9, 13). The higher digestibility of crude protein and total phosphorus observed in the low Ca–P group supports this mechanism. Moreover, high dietary Ca can bind fatty acids to form calcium soaps, which may interfere with lipid digestion and indirectly affect microbial fermentation of fibrous components in the gut (10). The markedly improved crude fiber digestibility observed in the low Ca–P group suggests that reducing dietary Ca may create a more favorable intestinal environment for microbial degradation of fiber. In addition, diets with high Ca levels possess greater buffering capacity, which may elevate gastric pH and impair pepsin activity, particularly in newly weaned piglets with immature digestive function (14). Therefore, lowering dietary Ca may enhance gastric protein digestion and contribute to improved overall nutrient utilization.

The relative stability of serum Ca and P concentrations reflects the strong homeostatic regulatory capacity of the organism. Serum alkaline phosphatase (ALP) activity was significantly elevated in the low Ca–P group, a response commonly regarded as an adaptive mechanism to low Ca intake (15). ALP plays a crucial role in bone formation and intestinal Ca and P transport, and its increased activity may indicate an upregulation of intestinal Ca absorption efficiency or bone mobilization rates to maintain calcium homeostasis (16). This interpretation is further supported by the significantly increased apparent digestibility of total calcium observed in this study. Notably, serum aspartate aminotransferase (AST) activity was significantly reduced in the low Ca–P group. AST is a sensitive indicator of hepatocellular damage and elevated metabolic burden (17). Excessive mineral intake requires increased hepatic and renal metabolism and excretion, thereby potentially increasing organ metabolic load (17). The reduction in AST activity suggests that a dietary Ca and P level of 0.60%/0.60% is more favorable for hepatic function in piglets and contributes to the maintenance of liver physiological health. In contrast, no significant changes were observed in GSH-Px, T-SOD, CAT, MDA, or T-AOC, indicating that the magnitude of Ca and P adjustment applied in this study did not induce oxidative stress. This finding is consistent with previous reports demonstrating that Ca and P nutrition primarily affects bone metabolism, intestinal function, and immune regulation, with limited direct impact on oxidative stress responses (2, 18).

Analysis of peripheral immune cells revealed that the low Ca–P group exhibited a 16.80% reduction in total white blood cell count and a 48.36% decrease in monocyte count, suggesting that reduced dietary Ca and P levels may influence peripheral immune cell reserves. Previous studies have established that Ca serves as a critical second messenger in intracellular signaling and is essential for immune cell activation and differentiation (19, 20). Insufficient Ca supply may therefore lead to impaired immune cell function or reduced circulating immune cell numbers (20). Moreover, the pronounced reduction in monocyte counts may indicate a suppressive effect of low Ca–P diets on systemic inflammatory and stress responses (21, 22). However, since serum Ca and inorganic phosphorus concentrations were not significantly altered in this study, these changes are more likely attributable to modifications in tissue-level Ca signaling and immune–metabolic interactions rather than to overt serum mineral deficiency.

Serum interleukin-10 (IL-10; anti-inflammatory cytokine) and interferon-γ (IFN-γ; pro-inflammatory cytokine) concentrations in the low Ca–P group decreased by 6.85 and 16.26%, respectively, indicating that reduced dietary Ca and P levels may lower the overall inflammatory tone of the organism. Previous studies have shown that Ca signaling is a key regulator of T-cell cytokine secretion, and reductions in Ca availability can simultaneously suppress cytokine production by both Th1 and regulatory *T* cells (19, 23). Therefore, the concurrent decline in IL-10 and IFN-γ observed in this study is consistent with established immunophysiological mechanisms. Notably, the decrease in IL-10 was relatively modest compared with the more pronounced reduction in IFN-γ, suggesting a stronger suppression of pro-inflammatory responses, which aligns with the observed decrease in monocyte counts. Although no significant differences were detected in CD4^+^ and CD8^+^ T-cell proportions between groups, numerically higher values were observed in the low Ca–P group (increases of 55.29 and 35.24%, respectively). This trend may indicate a compensatory adjustment of T-cell subsets aimed at maintaining immune homeostasis under low Ca–P conditions. Previous studies have reported that changes in mineral nutrition can modulate immune cell proportions by affecting thymic development or peripheral T-cell survival (24, 25). Although statistical significance was not achieved in the present study, these trends warrant further investigation.

This study has several limitations. First, only two dietary Ca-P levels were evaluated, and therefore the optimal dose-response relationship could not be determined. Second, bone mineralization, skeletal strength, intestinal microbiota, and mineral excretion were not measured. Finally, the experiment lasted 39 days, and the long-term effects of reduced Ca-P feeding on later growth stages remain unclear. Future studies should include multiple Ca-P gradients, bone and excretion measurements, and economic analyses under commercial conditions.

## 5 Conclusions

Under the conditions of this study, a dietary Ca and P level of 0.60%/0.60% was more advantageous than 0.85%/0.70% for weaned piglets. This dietary strategy alleviated the antagonistic effects of excessive Ca on nutrient digestion, significantly improved protein and mineral utilization, and contributed to a lower basal inflammatory state and reduced metabolic burden, while maintaining normal calcium–phosphorus homeostasis. These findings support the formulation of more precise and environmentally sustainable mineral nutrition strategies for weaned piglets.

## Data Availability

The original contributions presented in the study are included in the article/supplementary material, further inquiries can be directed to the corresponding author.
